# Discipline of Head, Heart, and Hand

**Published:** 1870-12

**Authors:** C. R. Butler


### DISCIPLINE OF HEAD, HEART, AND HAND.

BY C. R. BUTLER, D. D. S.

Read before the Ohio State Dental Society.

It might seem an act of supererogation to bring to the notice of this intelligent body that we lack discipline of head, heart, and hand, in performing the duties required at our hands. And yet the evidence comes to our apprehension day by day that such is the fact.

Let us notice first those that have been long in the way, in reference to the lack of discipline of the head: They commenced when the opportunities and facilities for thorough preparations for the duties, demands, and responsibilities were not what may be enjoyed to-day. Private tutors were few, colleges scarce, and of associations, none. And, as these facilities have been increased, comparatively few have made any marked degree of self-amelioration. Is it any wonder, then, that these give no support to our colleges or societies, by their money or personal influence? If we take them at their word, they are not indebted to dental schools, societies, or anybody else, for what they know. Nor can we expect them to give good, sound advice to a young man seeking a place as a student.

And just at this point, I fear many who have entered the profession more recently make grave mistakes, by taking students that can never be ornaments to the profession or a credit to their preceptors, who should be discouraged from entering the field of professional effort. We should take a good, firm position in such things and see that our fruits are a blessing to the profession and the world at large. I fear many that decry quacks are not able to say honestly that they did not have something to do in making them.

It is much easier to make a musician of a person that has innate musical talents, than of one that has but little or no music in his soul.

If we have no heart in the profession of our choice, the sooner we get out of it the better for all concerned.

We should have the kind of love for our work that will enable us to stand up like *men* and do our duty fearlessly, neither cringing nor begging of any one. Earnest and honest effort is sure to bring its reward.

We should be capable of meeting the demands that are made upon us; and it is within us to regulate the demand to a considerable extent, unless we are anxious to show our ignorance by publishing a guarantee to give satisfaction in all cases.

It is not necessary for a professional man to be anxious and always on the alert to bring himself before the people, and tell them what *they* ought to do, and, at the same time, that he is in want more than they are.

One of the first and most important lessons we should learn and never forget, is that the people need, and want our services, and will be much better satisfied if they are allowed to select who shall serve them. It is not complimentary, to say the least, for us to persist in electing for them.

The notion that some have, when they present themselves for dental operations, is that they are conferring a great favor on the Dentist by giving him a "*job*." The simple truth is, it should be a reciprocal thing on the part of both; any patronage or service rendered otherwise is not worth the having.

Another important item I would not fail to mention: if there be any present that have the notion that they have competitors in practice, they had better cast it aside as an unsightly garment, and arise to the manhood of their individuality, fully convinced that there is no rival outside their own operating room. There the war must be waged with self and disease; and he that can win daily victories there, will show that he has a disciplined head, heart and hands.

It is an individual work that belongs to each operator; and he should strive, as such, to take the cases as they *come* to him, and do the best his science and his skill will admit, to repair the effects of disease.

We may have the theories well stored in the head, and have the heart to do, but just the *how* to make the proper manipulation tests the most skillful.

Recognizing this all important item to successful practice, the question naturally presents itself how shall a student become skilled in the firm, yet delicate manipulations so essential to operative dentistry? Will you take him into your office, assign him to duty in the laboratory for three, six, or twelve months, scarcely allowing him in the operating room to see an impression taken, or a set of teeth adjusted to the parts that must surround them, or anything that pertains to adaptation and articulation, but simply work to the model you may give him, and possibly now and then filch a chance in your absence to extract a tooth, then send him out around the country to *practice*, saying, "If you will only persevere, a Dentist you will surely be." There can be no excusing circumstances for such a course.

Would it not be more manly and honest when a young man comes, desiring you to take him as a student, to tell him plainly that the profession needs men, not clowns; and if he be sufficiently earnest to take up the study of dentistry in all its branches, a reasonable length of time on his part, that you will strive to teach him faithfully the rudiments of practice, to be completed by his attending Lectures in a good Dental School, where not only the science of dentistry is taught, but where those principles are reduced to daily practice by and with a teacher that is capable of directing the hands in that discipline that will make them fit servants of a sound head and honest heart.

If the course be pursued that has just been hinted at, we may become possessed of a well disciplined head, heart, and hand.

